# LonP1 Differently Modulates Mitochondrial Function and Bioenergetics of Primary Versus Metastatic Colon Cancer Cells

**DOI:** 10.3389/fonc.2018.00254

**Published:** 2018-07-09

**Authors:** Lara Gibellini, Lorena Losi, Sara De Biasi, Milena Nasi, Domenico Lo Tartaro, Simone Pecorini, Simone Patergnani, Paolo Pinton, Anna De Gaetano, Gianluca Carnevale, Alessandra Pisciotta, Francesco Mariani, Luca Roncucci, Anna Iannone, Andrea Cossarizza, Marcello Pinti

**Affiliations:** ^1^Department of Medical and Surgical Sciences for Children and Adults, University of Modena and Reggio Emilia, Modena, Italy; ^2^Department of Life Sciences, University of Modena and Reggio Emilia, Modena, Italy; ^3^Department of Surgery, Medicine, Dentistry and Morphological Sciences, University of Modena and Reggio Emilia, Modena, Italy; ^4^Department of Morphology, Surgery and Experimental Medicine, Section of Pathology, Oncology and Experimental Biology and LTTA Center, University of Ferrara, Ferrara, Italy; ^5^Department of Diagnostic, Clinical Medicine and Public Health, University of Modena and Reggio Emilia, Modena, Italy

**Keywords:** LonP1, mitochondria, bioenergetics, beta-catenin, colorectal cancer, protease

## Abstract

Mitochondrial Lon protease (LonP1) is a multi-function enzyme that regulates mitochondrial functions in several human malignancies, including colorectal cancer (CRC). The mechanism(s) by which LonP1 contributes to colorectal carcinogenesis is not fully understood. We found that silencing LonP1 leads to severe mitochondrial impairment and apoptosis in colon cancer cells. Here, we investigate the role of LonP1 in mitochondrial functions, metabolism, and epithelial–mesenchymal transition (EMT) in colon tumor cells and in metastasis. LonP1 was almost absent in normal mucosa, gradually increased from aberrant crypt foci to adenoma, and was most abundant in CRC. Moreover, LonP1 was preferentially upregulated in colorectal samples with mutated p53 or nuclear β-catenin, and its overexpression led to increased levels of β-catenin and decreased levels of E-cadherin, key proteins in EMT, *in vitro*. LonP1 upregulation also induced opposite changes in oxidative phosphorylation, glycolysis, and pentose pathway in SW480 primary colon tumor cells when compared to SW620 metastatic colon cancer cells. In conclusion, basal LonP1 expression is essential for normal mitochondrial function, and increased LonP1 levels in SW480 and SW620 cells induce a metabolic shift toward glycolysis, leading to EMT.

## Introduction

LonP1 (also known as Lon or LonP) is one of the main proteases patrolling the mitochondrial matrix. Lon is a multi-function enzyme, exerting both proteolytic and chaperone activities, and also binds mitochondrial DNA and RNA [reviewed in Ref. (1)]. Main targets of LonP1 proteolytic activity are: (i) folded proteins, including 5-aminolevulinic acid synthase, steroidogenic acute regulatory protein, mitochondrial transcription factor A, and cytochrome c oxidase 4 isoform 1; (ii) misfolded proteins, including glutaminase C; (iii) oxidized proteins, including aconitase and cystathionine β-synthase (2–8). Conversely, targets of LonP1 chaperone activity are still not known. Initial studies demonstrated that LonP1 was involved in mitochondrial maintenance and mitochondrial quality control during aging (9, 10). More recent evidence suggests that LonP1 is responsible for additional functions critical to tumor progression, including metabolic adaptation to hypoxia, protection against senescence, and resistance to apoptosis and oxidative stress (11–14). Recent data from our group demonstrated that LonP1 is upregulated in colorectal tumors, and that its downregulation or inhibition leads to severe mitochondrial dysfunction and to apoptosis (15, 16).

Colorectal cancer (CRC) is the third most common cancer worldwide (17). Despite several advances have been made in the diagnosis and treatment of CRC over the past decades, the overall prognosis still remains poor, and tumor metastasis represent a major obstacle to successful treatment (18). The epithelial–mesenchymal transition (EMT) is a biologic process that enables a polarized epithelial cell to assume a mesenchymal cell phenotype through biochemical changes and a number of distinct molecular processes (19). Several intracellular signaling pathways contribute to EMT, and involve ERK, MAPK, PI3K, Akt, Smads, β-catenin (β-ctn), Ras, c-fos, and among others (19). The Wnt/β-ctn signaling pathway has a crucial role in the negative regulation of E-cadherin, and in the development of EMT and CRC metastasis (20). In normal conditions, and in the absence of activated Wnt signals, β-ctn is phosphorylated by APC/Axin/GSK-3β complex and then degraded by the proteasome. When Wnt ligands activate Frizzled and LPR receptors, β-ctn is no longer phosphorylated and translocates into the nucleus where it binds to transcription factors belonging to the family of T-cell factor (TCF) and lymphoid enhancer-binding protein, and then it activates transcription (21). In CRC, the vast majority of tumors have mutations in the key regulatory factors of the Wnt/β-ctn pathway and up to 80% present nuclear accumulation of β-ctn (20).

Starting from our previous observations, we aimed at investigating the precise role of LonP1 in colon primary tumor and metastasis. In this study, we show that elevated expression of LonP1 in CRC tissues is associated with nuclear localization of β-ctn, and that overexpression of LonP1, *in vitro*, differently affects β-ctn expression in SW480 and SW620 colon cancer cells. We also show that LonP1 overexpression differently affects mitochondrial functions and bioenergetics in SW480 and SW620 cells.

## Materials and Methods

### Cell Culture

Four cancer cell lines were used for this study: I407 intestinal epithelial cells, SW480 and RKO colon carcinoma cells, and SW620 metastatic colon cancer cells. SW480 cells were cultured in DMEM high glucose supplemented with 10% fetal bovine serum (FBS) and gentamycin. I407, RKO, and SW620 cells were cultured in RPMI Glutamax supplemented with 10% FBS and gentamycin. Cells were maintained in 5% CO_2_ atmosphere at 37°C. Culture media and reagents were from ThermoFisher Scientific (Eugene, OR, USA).

### Human Colorectal Tissues

We have studied a total of 45 patients who underwent surgical removal of CRC. Samples used for immunohistochemistry were formalin-fixed paraffin-embedded (FFPE) specimens, the other samples had been freshly frozen with a passage in liquid nitrogen, and then stored at −80°C until use. Tissues were obtained from the Department of Diagnostic and Clinical Medicine, and Public Health, University of Modena and Reggio Emilia, through an institutional review board-approved protocol. Demographic and clinical characteristics of patients are reported in Tables 1 and 2.

**Table 1:** Demographic characteristics and tumor stage in samples obtained from NM, ACF, Ad, or CRC.

Patient	Stage	Age	Sex
1	NM	84	M
2	NM	85	M
3	NM	78	M
4	NM	86	M
5	NM	84	M
6	NM	46	M
7	NM	52	F
8	NM	82	M
9	NM	46	F
10	NM	86	M
11	NM	43	M
12	NM	82	M
13	ACF	38	F
14	ACF	43	M
15	ACF	46	F
16	ACF	38	F
17	ACF	43	M
18	ACF	43	M
16	Ad	38	F
17	Ad	43	M
18	Ad	46	F
19	Ad	46	F
20	Ad	43	M
21	Ad	43	M
22	CRC	85	M
23	CRC	86	M
24	CRC	46	F

*M, male; F, female; NM, normal mucosa; ACF, aberrant crypt foci; Ad, adenoma; CRC, colorectal cancer*.

**Table 2:** Demographic characteristics, tumor stage, Ki-67, β-catenin (β-ctn), E-cadherin (E-cad), p53, LonP1 expression in 21 colorectal cancer samples.

Patient	Age	Sex	Stage	Site	Ki-67 (%)	β-ctn	E-cad	p53	Lon IHC	LonP1 Wb
1	73	M	II	Ascending	90	N	P	+	>75%	0.92
2	75	M	II	Rectum	60	N	P	+	>75%	4.06
3	55	F	I	Rectum	60	N	P	+	>75%	3.45
4	61	F	III	Rectum	50	Me	P	+	>75%	2.10
5	59	F	II	Sigmoid	40	N	P	+	>75%	76.70
6	61	F	III	Rectum	60	Me	P	+	>75%	4.94
7	83	F	III	Rectum	60	Me	P	−	>75%	1.37
8	66	F	I	Rectum	80	N	P	+	>75%	35.30
9	81	F	III	Ascending	70	Me	P	+	30–75%	48.85
10	63	M	IV	Ascending	40	Me	P	+	<30%	0.27
11	72	M	III	Ascending	60	Me	P	−	30–75%	2.55
12	88	M	I	S-R	70	Me	P	−	<30%	0.64
13	80	M	IV	S-R	40	N	P	−	<30%	3.60
14	71	F	II	S-R	40	N	P	−	30–75%	2.53
15	71	M	IV	Rectum	80	N	P	−	30–75%	1.17
16	47	M	IV	S-R	80	N	P	+	30–75%	7.47
17	62	F	IV	Ascending	50	Me	P	−	<30%	0.16
18	73	F	I	Rectum	70	N	P	+	30–75%	3.56
19	60	F	IV	Rectum	40	Me	P	+	<30%	0.10
20	56	M	III	Rectum	60	N	P	−	<30%	0.29
21	75	F	I	Rectum	70	Me	P	−	30–75%	1.04

*M, male; F, female; Me, membrane; N, nuclear; P, present*.

### Retroviral Transduction

The pMSCV-Puro empty vector and the pMSCV containing the cDNA encoding for Lon protease (hereafter referred to as pLonP1) were used to transiently transfect amphotrophic Phoenix cell line (15). Cells infected with the empty vector will be indicated as pMSCV cells. Retroviral supernatants were used to stably transfect I407, RKO, SW480, and SW620 cells, and stable transfectants were selected by using 3–4 µg/ml puromycin (depending on the cell line), and then maintained in cell medium supplemented with 2 µg/ml puromycin.

### RNA Interference

Cells were reverse transfected by using RNAiMAx (Life Technologies Corporation) and 10 nM s17901 small interfering RNAs (Life Technologies Corporation) against LonP1 mRNA. Then, cells were incubated for 72 h, trypsinized, and lysated by using RIPA buffer.

### Immunohistochemical Analyses

Tumor specimens were taken from patients who underwent surgical resection of the large bowel in the period 2010–2015. All slides were blindly reviewed by the pathologist (LL). For each case, a representative paraffin-embedded block containing tumor tissue and normal mucosa, as internal control, was sectioned at 4 µm. Immunoperoxidase staining was run with the Benchmark XT Automatic Staining System (Ventana Roche) with diaminobenzidine as chromogen and using the View DAB Detection Kit (Roche). At the end of the reaction, slides were counterstained with hematoxylin. The following antibodies were used: mouse monoclonal antibodies β-ctn (Roche, Basilea, Switzerland), p53 (Roche), and E-cadherin (Dako, Santa Clara, CA, USA) available as pre-diluted commercially preparations; mouse monoclonal Ki-67 antibody (MIB-1, Dako, used at 1:100 dilution), and rabbit polyclonal LonP1 antibody (Primm, Milan, Italy, used at a 1:500 dilution). According to previous reports, expression of β-ctn and E-cadherin in normal colon epithelium resulted to be restricted to cell membrane. Altered expression of β-ctn was contemplated when 10% of tumor cells or greater showed nuclear or cytoplasmic immunoreactivity. Loss of membrane expression of E-cadherin was considered in cases exhibiting either no immunoreactivity or 10% of tumor cells or less with positive membranous staining. Detection of proliferative activity was carried out using an anti-Ki-67 antibody, and basal cells of the normal colonic crypts were used as internal positive control. Ki-67 labeling index was determined by counting the number of positive nuclei for 1,000 neoplastic cells in 10 consecutive fields chosen randomly in non-necrotic areas of the tumor.

### Immunoblotting

Total protein lysate was obtained from cell lines by using RIPA buffer and from FFPE tissue as previously described (22). Protein concentration was determined by Bradford assay. Proteins were then separated in 4–12% or 12% Bolt Bis-Tris precast gels (Thermo Fisher Corporation) and transferred onto nitrocellulose membranes. Protein transfer was performed by using methanol transfer buffer or by using Trans-Blot Turbo cassettes (Bio-Rad Laboratories) with Trans-Blot Turbo blotting system (Bio-Rad Laboratories). Rabbit polyclonal anti-LonP1 was a custom antibody from Primm (Milan, Italy). Rabbit polyclonal anti-β-actin and rabbit polyclonal anti-E-cadherin were from Abcam (Cambridge, UK). Rabbit polyclonal to β-ctn, to lactate dehydrogenase A (LDHA), to glucose 6-phosphate dehydrogenase (G6PD), to N-cadherin, to Akt, to phospho-Akt (Ser473), to GSK-3β, to phospho-GSK-3β (Ser9), and to Twist were from Cell Signaling Technology (Danvers, MA, USA). Images were acquired by using ChemiDoc MP (Bio-Rad Laboratories) and Image Lab version 5.2.1 was used to perform densitometric analysis.

### Immunofluorescence

Cells were grown on coverslips and fixed for 15 min in PBS containing 4% paraformaldehyde. Samples were then washed three times with PBS, incubated in PBS containing 0.1% Triton X-100 for 1 h at room temperature, and blocked by using PBS containing 3% bovine serum albumin for 1 h. Samples were then incubated with PBS containing primary antibody and secondary antibodies for 60 and 30 min, respectively. The following antibodies were used: rabbit polyclonal anti-β-ctn (Cell Signaling Technology) and anti-rabbit Alexa Fluor 647. Images were acquired by using a Nikon A1 confocal laser scanning microscope (Nikon, Tokyo, Japan).

### Transmission Electron Microscopy

Cell pellets were fixed in 2.5% glutaraldehyde in Sorensen’s Phosphate Buffer 0.1 M pH 7.4 (PB) for 1 h and post-fixed in 1% OsO_4_ in PB 0.1 M for 1 h, as previously described (23). Then, ultrathin sections were cut from Durcupan embedded samples, collected on nickel grids, stained with uranyl acetate and lead-citrate, and then analyzed by using a Zeiss EM 109 Transmission Electron Microscope (Zeiss AG, Jena, Germany).

### Oxygen Consumption Rate (OCR)

The rate of oxygen consumption was assayed with the XF96 Extracellular Flux Analyzer (Seahorse Biosciences—Agilent Technologies, Santa Clara, CA USA). Cells were plated 2 days before the experiment, and experiments were performed on a confluent monolayer. The number of cells that were plated was: 5 × 10^4^/well for SW620-pMSCV and SW620-pLonP1, and 4 × 10^4^/well for SW480-pMSCV and SW480-pLonP1.

### Cytofluorimetric Analyses

Flow cytometry was used to determine mitochondrial mass, mitochondrial membrane potential (MMP), mitochondrial reactive oxygen species (ROS), and glucose transporter (GLUT)-1 expression. For mitochondrial mass analysis, cells were incubated with MitoTracker Green FM (MTG, 200 nM, Thermo Fisher Corporation) for 30 min at 37°C. For MMP analysis, cells were incubated with tetramethylrhodamine, methyl ester (TMRM, 200 nM, Thermo Fisher Corporation) for 10 min at 37°C. Carbonyl cyanide m-chlorophenyl hydrazine (CCCP, 1 and 10 µM, Sigma Aldrich) was used to induce mitochondrial membrane depolarization. Mitochondrial superoxide production was assessed using MitoSox Red Mitochondrial Superoxide Indicator (mtSOX, 5 µM, ThermoFischer Corporation). GLUT-1 expression was assessed by staining cells with anti-GLUT-1 Alexa Fluor 488 (Abcam). Data were collected on an Attune NxT (Thermo Fisher Corporation) and analyzed with FlowJo software (Tree Star, Ashland, OR, USA).

### Statistical Analysis

Quantitative variables were compared with non-parametric Mann–Whitney test. Statistical analyses were performed using GraphPad 5.0 (Prism, La Jolla, CA, USA). Error bars represent SD. A *p* value <0.05 was considered significant.

## Results

### LonP1 Is Upregulated in CRC and Is Associated With Mutated p53 or Nuclear β-ctn

**Figure 1:**
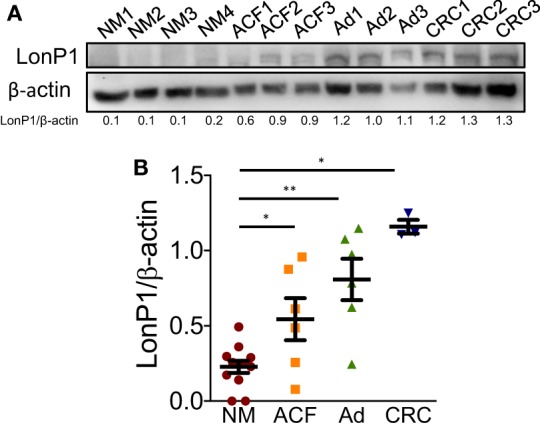
LonP1 expression during colon cancer progression. **(A)** Representative Western blot analysis of LonP1 expression in freshly frozen tissue samples from normal mucosa (NM), aberrant crypt foci (ACF), colonic adenoma (Ad), and colorectal cancer (CRC). **(B)** Relative protein level of LonP1 in NM, ACF, Ad, and CRC. Data are reported as mean ± SD; *P < 0.05 and **P < 0.01.

**Figure 2:**
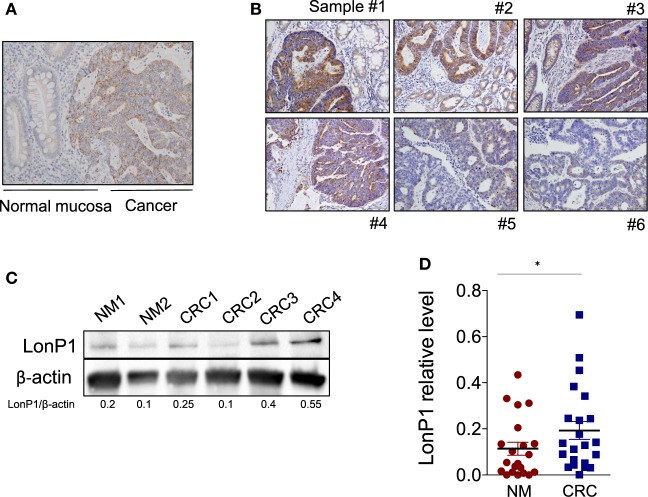
LonP1 is upregulated in CRC tissues. **(A)** Representative immunohistochemical staining of LonP1 in paraffin-embedded human colorectal cancer (CRC) tissue and patient-matched adjacent normal mucosa. **(B)** Representative immunohistochemical stainings of LonP1 high or low level overexpression in paraffin-embedded human CRC tissues. **(C)** Representative Western blot analysis of LonP1 expression in CRC tissues and normal mucosa. **(D)** Relative protein levels of LonP1 are significantly higher in CRC tissues than in adjacent normal mucosa. Data are reported as mean ± SD (*n* = 21); *P < 0.05.

We previously demonstrated that LonP1 silencing is associated with severe mitochondrial dysfunction and apoptosis susceptibility in colon cancer cell lines (22). Herein, we quantified the expression of LonP1 during colon cancer progression in fresh frozen tissues of ACF, adenoma (Ad), and CRC from a total of 24 patients, whose demographic and clinical characteristics are reported in Table 1. We found that the LonP1 was almost absent in normal mucosa, gradually increased from samples of ACF to Ad, and was most abundant in samples of CRC (Figures 1A,B). In addition, we quantified LonP1 levels in FFPE samples from 21 patients affected by CRC, whose demographic and clinical characteristics are reported in Table 2. Despite inter-individuals variations, LonP1 was upregulated in CRC samples compared with the normal mucosa counterpart (Figures 2A–D).

**Figure 3:**
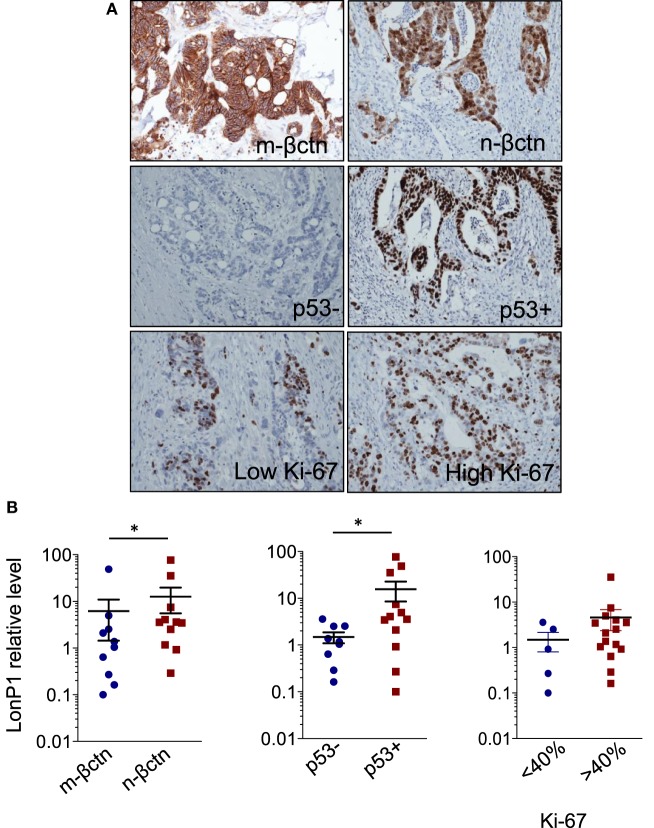
LonP1 upregulation is associated with mutated p53 or nuclear β-ctn. **(A)** Representative immunohistochemical stainings of Ki-67, membrane β-ctn (m-βctn), nuclear β-ctn (n-βctn), and p53 in colorectal cancer (CRC) tissues. **(B)** Association between expression of LonP1, mutated p53, non-mutated p53, membrane β-ctn, or nuclear β-ctn in CRC tissues; *P < 0.05.

In the same FFPE samples, we explored the expression levels of EMT-related proteins together with mutated p53 and Ki-67, and their association with LonP1. Representative images of immunohistochemical staining are reported in Figure 3A. We found that LonP1 was preferentially upregulated in colorectal samples with mutated p53 or nuclear β-ctn (Figure 3B). No differences were observed regarding Ki-67 levels.

### LonP1 Modulation Is Associated With Changes in β-ctn Levels

**Figure 4:**
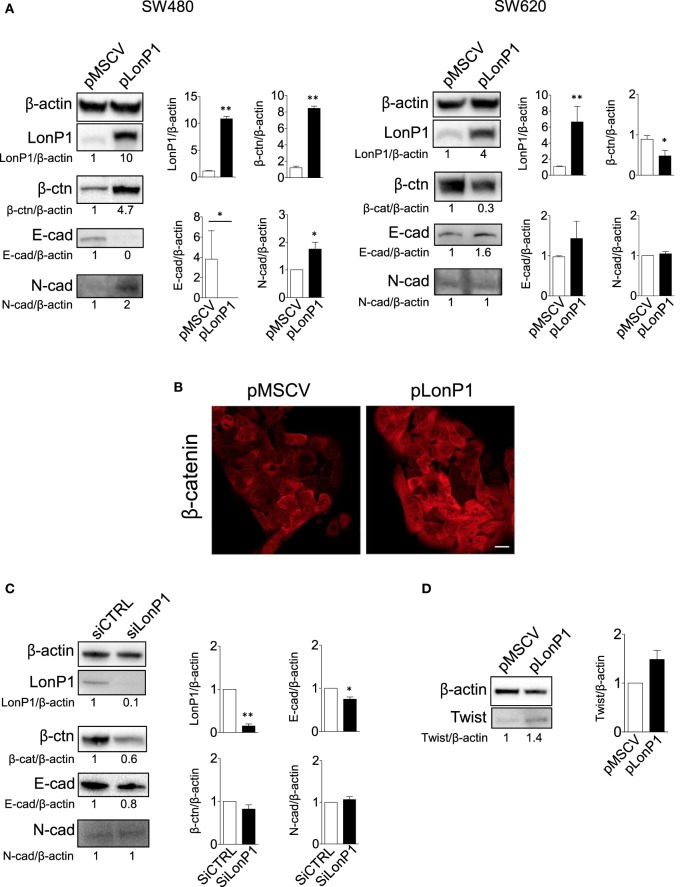
LonP1 modulates E-cadherin, N-cadherin, and β-ctn in SW480 colon primary tumor cells and SW620 metastatic tumor cells. **(A)** Representative Western blot analysis and relative protein level of LonP1, β-catenin (β-ctn), E-cadherin (E-cad), N-cadherin (N-cad) in SW480 and SW620 cells overexpressing LonP1 (namely pLonP1) and in control cells, i.e., cells stably transfected with empty vector (namely pMSCV). β-actin was used as loading control. Densitometries are reported in histograms, and data are reported as mean ± SD (*n* = 3). *P < 0.05 and **P < 0.01. **(B)** Representative confocal microscopy image showing β-ctn localization in SW480-pMSCV and SW480-pLonP1 cells. Bar, 10 μm. **(C)** Representative Western blot analysis and relative protein level of LonP1, β-ctn, and E-cad SW620, where LonP1 has been downregulated by using small interfering RNAs (siRNAs) against LonP1 mRNA (siLonP1). Control cells were transfected with scramble siRNAs and are indicated as siCTRL. Densitometries are reported in histograms, and data are reported as mean ± SD (*n* = 3). *P < 0.05 and **P < 0.01. **(D)** Representative Western blot analysis and relative protein level of Twist in SW480 cells overexpressing LonP1 (namely pLonP1) and in control cells, i.e., cells stably transfected with empty vector (namely pMSCV). β-actin was used as loading control. Densitometries are reported in histograms, and data are reported as mean ± SD (*n* = 3).

To investigate the link between LonP1 and EMT in colon, we took advantage of four different cell lines, I407, RKO, SW480, and SW620 cells. I407 are intestinal epithelial immortalized cells. RKO and SW480 are colon carcinoma cells, whereas SW620 are metastatic colon cancer cells. Moreover, SW480 and SW620 represent a primary adenocarcinoma tumor and a lymph node metastasis from the same patient, respectively. We first established stable LonP1 overexpression in SW480 cells, and quantified the levels of several EMT-related proteins, including β-ctn, E-cadherin, and N-cadherin. Forced LonP1 expression led to increased β-ctn levels, reduced E-cadherin, and increased N-cadherin in primary adenocarcinoma cells SW480 (Figure 4A, left panels). Similar results were obtained by upregulating LonP1 in I407 and RKO cells (Figures S1A,C in Supplementary Material). In particular, forced LonP1 expression in these cells led to threefold decrease of E-cadherin levels and 2.5-fold increase of β-ctn levels in I407 and RKO cells, respectively. I407-pLonP1 cells also exhibited an elongated, mesenchymal-like morphology, which is typically observed in EMT (Figure S1B in Supplementary Material). Modulation of β-ctn in SW480-pLonP1 cells was also confirmed by fluorescence microscopy (Figure 4B). The upregulation of LonP1 in SW620 cells, which are metastatic cells, led to opposite results (Figure 4A, right panels). Interestingly, downregulation of LonP1 in SW620 cells was associated with reduced β-ctn expression, increased E-cadherin expression, and unchanged N-cadherin in SW620 cells (Figure 4C). Considering that Twist is a key regulator of EMT, its expression was analyzed in SW480 cells overexpressing LonP1. Twist levels were almost twofold increased in SW480-pLonP1 cells, if compared to control SW480-pMSCV cells (Figure 4D).

**Figure 5:**
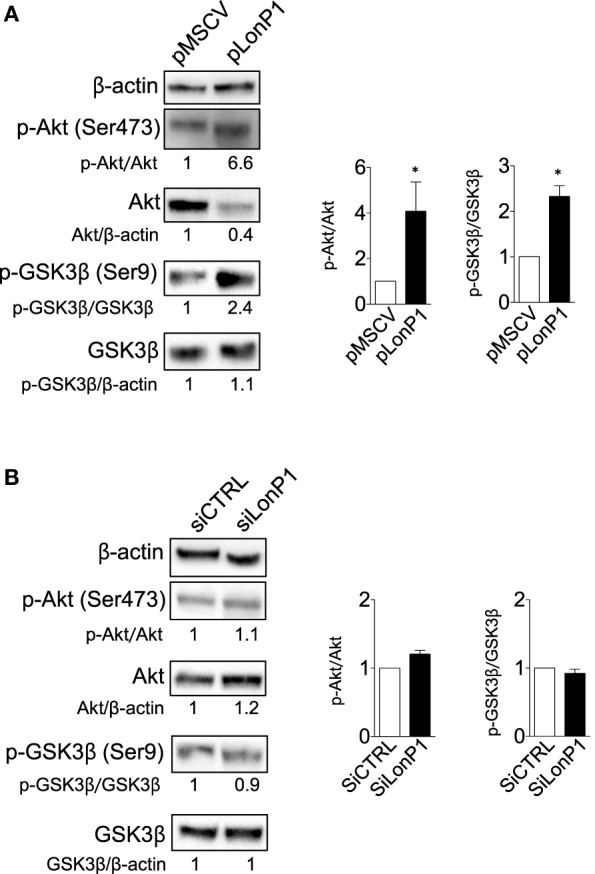
LonP1 modulates β-ctn by regulating Akt/GSK3β signaling. **(A)** Representative Western blot analysis and relative protein levels of phosphorylated Akt (Ser473), Akt, phosphorylated GSK-3β (Ser9), and GSK-3β in SW480 cells overexpressing LonP1. **(B)** Representative Western blot analysis and relative protein levels of phosphorylated Akt (Ser473), Akt, phosphorylated GSK-3β (Ser9), and GSK-3β in SW620 downregulating LonP1. β-actin was used as loading control. Densitometries are reported in histograms, and data are reported as mean ± SD (*n* = 3). *P < 0.05.

Our study confirmed that LonP1 modulation is associated with changes in β-ctn levels and distribution, both *in vitro* and *ex vivo*, in samples from CRC patients. Considering the central role of Akt and GSK-3β in the regulation of β-ctn, we examined the phosphorylation status of Akt and GSK-3β, at serine 473 and serine 9, respectively. We found that LonP1 overexpression led to increased levels of phosphorylated Akt (p-Akt) and phosphorylated GSK-3β (p-GSK-3β), whereas LonP1 depletion led to a slight decrease in p-Akt and p-GSK-3β (Figures 5A,B).

### LonP1 Impacts Glycolysis in Colon Cancer Cells

**Figure 6:**
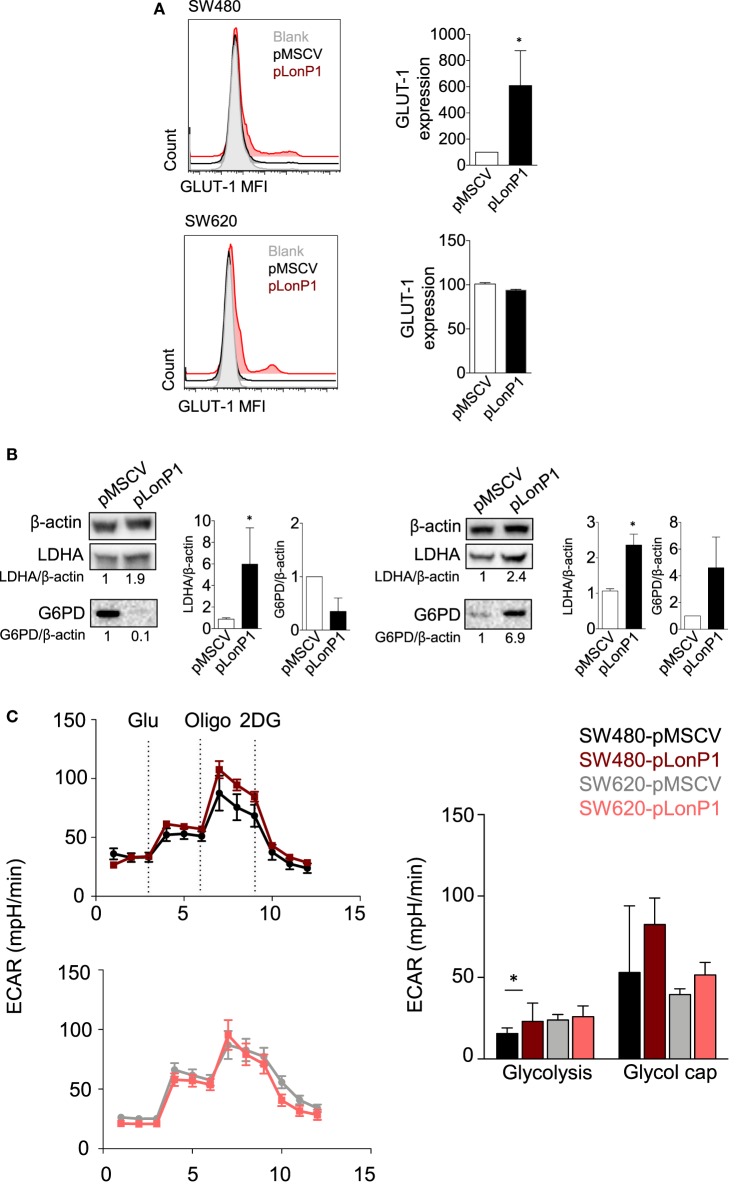
LonP1 modulates glycolytic activity in colon cancer cells. **(A)** Representative histograms showing glucose transporter (GLUT)-1 expression in SW480-pMSCV, SW480-pLonP1, SW620-pMSCV, and SW620-pLonP1 cells. Quantitative analysis is reported on the right and shows the median fluorescence intensity of plasma membrane GLUT-1 levels, as revealed by flow cytometry. **(B)** Representative Western blot analysis and relative protein levels of phosphorylated lactate dehydrogenase A and glucose 6-phosphate dehydrogenase in SW480 and SW620 cells overexpressing LonP1. β-actin was used as loading control. Densitometries are reported in histograms, and data are reported as mean ± SD (*n* = 3). *P < 0.05. **(C)** Representative traces and quantitative analysis of extracellular acidification rate in indicated cells. Subsequent injections of glucose (Glu), oligomycin (Oligo), and 2-deoxy-glucose were performed as indicated.

As Akt/GSK-3β signaling pathway plays a critical role in regulating glucose metabolism (24), in SW480 and SW620 cells we examined whether forced LonP1 expression had effects on the expression of GLUT-1, LDHA, and G6PD. Plasma membrane GLUT-1 levels increased in SW480 cells overexpressing LonP1 (Figure 6A). LDHA levels increased whereas G6PD was almost undetectable in SW480 cells overexpressing LonP1 (Figure 6B). In this model, glucose 6-phosphate is likely converted into fructose 6-phosphate, thus entering the glycolytic pathway, rather than being converted into glucose 6-phosphate-gluconolactone, and entering the pentose phosphate pathway. In SW620 cells, both LDHA and G6PD levels increased in the presence of high levels of LonP1. We, therefore, analyzed the glycolytic activity of these cells by performing a real-time analysis of the extracellular acidification rate (ECAR, Figure 6C). SW480-pLonP1 cells exhibited a higher maximal glycolysis compared with SW480-pMSCV control cells. Quantitative analysis showed no significant differences in basal glycolysis. No changes were observed in SW620 cells.

**Figure 7:**
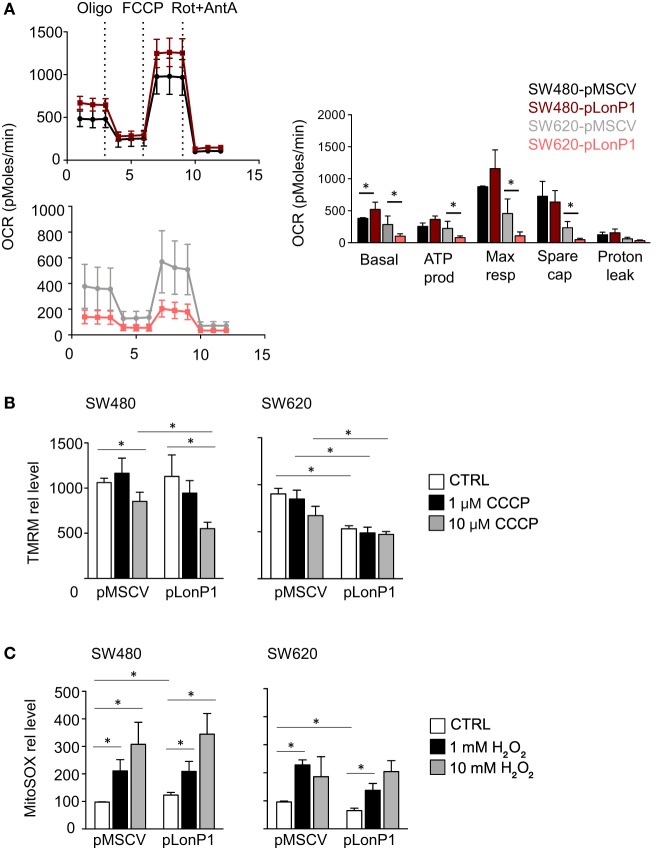

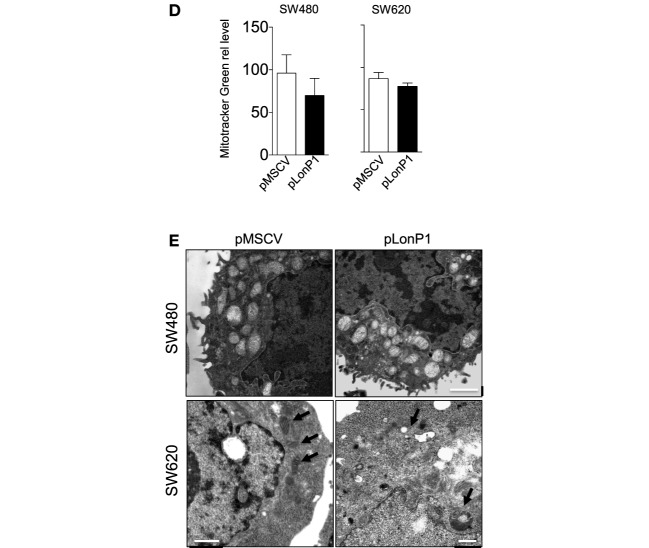
LonP1 slightly modulates mitochondrial activity in colon cancer cells. **(A)** Representative traces and quantitative analysis of the oxygen consumption rate in indicated cells. Subsequent injections of oligomycin (Oligo), mitochondrial decoupler carbonyl cyanide-4-(trifluoromethoxy)phenylhydrazone (FCCP), complex I inhibitor rotenone (Rot), and complex III inhibitor (AntA) were performed as indicated; *P < 0.05. **(B)** Mitochondrial membrane potential quantification was assayed by tetramethyl rodhamine methyl esther (TMRM) in the presence or absence of CCCP in indicated cells. Data are expressed as percentage of increase in median fluorescence intensity (MFI) and represented the mean ± SD (*n* = 4); *P < 0.05. **(C)** Mitochondrial anion superoxide quantification as assayed by MitoSOX Red Mitochondrial Superoxide Indicator (mitoSOX) in the presence or absence of hydrogen peroxide in indicated cells. Data are expressed as percentage of increase in MFI and represented the mean ± SD (*n* = 4). **(D)** Mitochondrial mass quantification as assayed by Mitotracker Green in indicated cells. Data are expressed as percentage of increase in MFI and represented the mean ± SD (*n* = 4); *P < 0.05. **(E)** Representative transmission electron microscopy images of indicated cells. Scale bars, 1 µm.

Considering that LonP1 has been involved in controlling tumor bioenergetics by reprogramming mitochondrial functions, we analyzed mitochondrial activity by monitoring OCR. Representative curves showed that a difference in mitochondrial respiration is present between SW480-pLonP1 and control cells (Figure 7A); quantitative analysis showed that basal OCR was higher in SW480-pLonP1 cells. Basal and coupled respiration as well as maximal respiration and spare respiratory capacity were lower in SW620-pLonP1 cells than in SW620-pMSCV cells (Figure 7A). Concerning OCR, slight differences were observed in I407 or RKO cells in the presence of higher levels of LonP1 (Figure S2A in Supplementary Material). Since MMP is required for production of ATP during oxidative phosphorylation (OXPHOS), we analyzed MMP in these cells, by using TMRM. MMP was unchanged in SW480-pLonP1 and I407-pLonP1 cells (Figure 7A; Figure S2B in Supplementary Material). However, both SW480-pLonP1 and control cells were sensitive to CCCP-induced MMP depolarization (Figure 7B). Interestingly, SW620-pLonP1 and RKO-pLonP1 cells had depolarized mitochondria if compared to SW620-pMSCV and RKO-pMSCV cells, respectively (Figure 7B; Figure S2B in Supplementary Material). The reduction of MMP in SW620-pLonP1 was maintained when cells were treated with 10 µM CCCP. As mitochondrial activity is a critical source of ROS, and in particular of anion superoxide, we analyzed its levels by using MitoSOX Red Mitochondrial Superoxide indicator (MitoSOX). We found that SW480-pLonP1 cells produced higher levels of anion superoxide compared to control cells, at the basal level (Figure 7C). When challenged with hydrogen peroxide (H_2_O_2_), no differences were observed between cells overexpressing LonP1 and control cells (Figure 7C; Figure S2C in Supplementary Material). Mitochondrial superoxide decreased in SW620-pLonP1 if compared to SW620-pMSCV control cells, both at the basal level and after treatment with H_2_O_2_ as pro-oxidant stressor (Figure 7C).

Finally, we asked whether LonP1 modulation had impact on mitochondrial mass and mitochondrial ultrastructure. Mitochondrial mass was slightly decreased in pLonP1 cells (Figure 7D). Analysis of mitochondrial ultrastructure revealed that in SW480-pMSCV and SW480-pLonP1 cells mitochondria were numerous, occupied most of the cytoplasm and displayed *cristae* fragmentation together with the presence of vacuoles and vesicles within the mitochondrial matrix (Figure 7E). SW620-pLonP1 cells displayed mitochondrial alterations to a much lesser extent, both in number and in shape, with reduced *cristae* and increased vacuoles, and a noteworthy abundance of free ribosomes in the cytoplasm (Figure 7E), reasonably due to a massive synthesis of endogenous proteins, which correlates with particularly aggressive and undifferentiated neoplasms. The counterpart SW620-pMSCV cells did not show altered morphology, or number of mitochondria, whose *cristae* appeared preserved (Figure 7E).

## Discussion

Several studies have detected frequent alterations in the expression of mitochondrial proteases in a variety of human tumors, suggesting that these proteins may play a role as a novel class of tumor promoters or suppressors (14, 25–28). LonP1 is a mitochondrial protease and chaperone located in the mitochondrial matrix. Initial studies placed LonP1 among “stress response proteins,” that is those proteins upregulated in response to cell stress (29, 30). More recently, LonP1 has been implicated in the control of metabolic networks in mitochondria in melanoma cells (14), and in hypoxic adaptation in glioma cells (11). We previously showed that: (i) LonP1 is upregulated in several cancer cell lines, including RKO, and in CRC tissues if compared to adjacent normal mucosa, and that (ii) colon cancer cells with low levels of LonP1 displayed reduced levels of OXPHOS complexes, reduced OCR, and increased mitochondrial ROS and highly fragmented and altered mitochondria (22). However, the precise role of LonP1 in colon cancer progression has not been clarified.

In this study, we analyzed the expression of LonP1 in different stages of colon cancer and the consequences of its upregulation at the mitochondrial and cellular level. The first observation is that LonP1 expression gradually increases in normal mucosa, ACF, Ad, and CRC. The fact that LonP1 levels are higher in CRC could suggest a potential role for LonP1 in EMT, an early step in the formation of metastasis (31). We observed high levels of LonP1 in CRC tissues with nuclear localization of β-ctn or in CRC tissues with mutated p53. It is noteworthy that both β-ctn and p53 are directly involved in EMT: β-ctn/TCF4 complex induces EMT through transcription activation of ZEB1 (32), whereas p53 regulates the transcription of genes that are involved in pathways that suppress tumor metastasis, and mutations of p53 can precede metastasis (33). Although previous studies have reported that LonP1 overexpression is associated with decreased E-cadherin, increased N-cadherin and vimentin, a possible link between LonP1 and β-ctn has never been reported (14, 34).

β-ctn is a key component of the Wnt signaling pathway, and acts as negative regulator of E-cadherin in the induction of EMT. In the absence of Wnt stimulation, cytoplasmic β-ctn is phosphorylated by the APC/Axin/GSK-3β complex, and is degraded into the proteasome (35). We reported that LonP1 modulation led to important changes in total β-ctn levels, in several colon cancer cell lines. To understand which pathway was involved in LonP1 regulation of β-ctn, we investigated the phosphorylation status of Akt and GSK-3β, as Akt can phosphorylate, and thus inactivate GSK-3β (36). To explore the role of LonP1 in Akt/GSK-3β signaling pathway, SW480 and SW620 colon carcinoma cell lines were chosen as models since they are derived from primary and secondary tumors resected from the same patient, that makes them a valid tool to investigate changes in colon cancer progression (37). In SW480 cells, the upregulation of an oncoprotein or a protein-like LonP1 would lead to EMT. On the contrary, the downregulation of the same protein in SW620 cells, which are already metastatic, would leave unchanged the mesenchymal phenotype. Indeed, forced LonP1 expression in SW480 led to increased levels of mesenchymal markers, and LonP1 upregulation was associated with increased phosphorylation of Akt and GSK-3β, thus highlighting a role for LonP1 as a regulatory factor in the Wnt/β-ctn pathway. Accordingly, it was interesting to observe that Twist, a key promoter of cancer progression, was involved in EMT. A number of studies have suggested that Twist induces EMT *via* AKT/GSK-3β/β-catenin pathways, among others (38).

While previous studies regarding LonP1 and cancer reported that LonP1 induced EMT through ROS-dependent MAPK signaling, at least in 293 T cells, we found that mitochondrial ROS were not implicated in EMT induction (34). Rather, other mechanisms could be involved, including the alteration of pathways involved in the rearrangement of cellular metabolism. It has been reported that LonP1 controls tumor bioenergetics by remodeling subunits of electron transport chain (14). We found that in colon cancer cells LonP1 can influence glycolytic activity together with mitochondrial activity. Alongside, effects of LonP1 overexpression have been observed in the initial steps of pentose phosphate pathway, which is required for the synthesis of ribonucleotides from glucose and is a major source of nicotinamide adenine dinucleotide phosphate. Pentose pathway is important for redox balance and anabolism, and seems indeed to be promoted in SW620-pLonP1 cells. In SW620-pLonP1 cells, glucose could be redistributed to alternative pathways, such as the pentose pathway, to support growth and survival of these cells, which were characterized by reduced OXPHOS. In agreement with this observation, the pentose phosphate pathway was strongly decreased in SW480-pLonP1, where OXPHOS was almost unchanged. Despite the fact that SW480 and SW620 have the same genetic background, characterized by mutations in KRAS and TP53, overexpression of LonP1 has dramatically different consequences on mitochondrial function, bioenergetics, and in malignant transformation. While LonP1 does not influence OCR or mitochondrial functions in SW480 cells, its overexpression in SW620 cells determines a reduction of OCR and depolarization of mitochondrial membrane. We would expect that mitochondrial membrane depolarization resulted in increased ROS production, but this was not case. We rather observed a decrease in mitochondrial anion superoxide in SW620-pLonP1 when compared with SW620-pMSCV cells. The analysis of morphology and ultrastructure of mitochondria confirmed that overexpression of LonP1 has a stronger impact on SW620 than SW480 cells.

In conclusion, our findings demonstrate a role for LonP1 in the regulation of EMT *via* GSK-3β/β-ctn and modifications in cellular metabolism, suggesting that changes in LonP1 expression and EMT in CRC are likely not two independent, concurrent phenomena, but might be functionally linked.

## Ethics Statement

This study was carried out in accordance with the recommendations of the Ethical Committee of the province of Modena (Italy). The protocol was approved by the Ethical Committee of the province of Modena. All subjects gave written informed consent in accordance with the Declaration of Helsinki.

## Author Contributions

LG, AC, and MP conceived and designed the experiments. LG, SB, DT, GC, AP, AG, SPe, and SPa conducted the experiments. LL, FM, and LR provided samples and performed immunohistochemical stainings. MN reported and organized data. LG, LL, AI, PP, AC, and MP wrote and revised the paper.

## Conflict of Interest Statement

The authors declare that the research was conducted in the absence of any commercial or financial relationships that could be considered as potential conflict of interest.

## Supplementary Material

Figure S1 LonP1 modulates E-cadherin, N-cadherin, and β-ctn in I407 and RKO cells. **(A)** Representative Western blot analysis and relative protein level of LonP1, β-catenin (β-ctn), E-cadherin (E-cad), N-cadherin (N-cad) in I407 cells overexpressing LonP1 (namely pLonP1) and in control cells, i.e., cells stably transfected with empty vector (namely pMSCV). β-actin was used as loading control. Densitometries are reported in histograms, and data are reported as mean ± SD (*n* = 3). **P* < 0.05 and ***P* < 0.01. **(B)** Representative phase contrast images of I407 cells overexpressing LonP1 (pLonP1) and control cells (pMSCV). Scale bar, 10 µm. **(C)** Representative Western blot analysis and relative protein level of LonP1, β-catenin (β-ctn), E-cadherin (E-cad), and N-cadherin (N-cad) in RKO cells overexpressing LonP1 (namely pLonP1) and in control cells, i.e., cells stably transfected with empty vector (namely pMSCV). β-actin was used as loading control. Densitometries are reported in histograms, and data are reported as mean ± SD (*n* = 3). **P* < 0.05 and ***P* < 0.01.

Figure S2 LonP1 slightly modulates mitochondrial activity in I407 and RKO colon cancer cells. **(A)** Representative traces and quantitative analysis of the oxygen consumption rate in indicated cells. Subsequent injections of oligomycin (Oligo), mitochondrial decoupler carbonyl cyanide-4-(trifluoromethoxy)phenylhydrazone (FCCP), complex I inhibitor rotenone (Rot) and complex III inhibitor (AntA) were performed as indicated. **(B)** Mitochondrial membrane potential quantification as assayed by tetramethyl rodhamine methyl esther (TMRM) in the presence or absence of CCCP in indicated cells. Data are expressed as percentage of increase in median fluorescence intensity (MFI) and represented the mean ± SD (*n* = 3); **P* < 0.05. **(C)** Mitochondrial anion superoxide quantification as assayed by MitoSOX Red Mitochondrial Superoxide Indicator (mitoSOX) in the presence or absence of hydrogen peroxide in indicated cells. Data are expressed as percentage of increase in MFI and represented the mean ± SD (*n* = 3); **P* < 0.05.
