# Supplementary figures and images for: LonP1 Differently Modulates Mitochondrial Function and Bioenergetics of Primary Versus Metastatic Colon Cancer Cells
Source: Front Oncol. 2018 Jul 9;8:254. doi: 10.3389/fonc.2018.00254 (PMC6046640; doi:10.3389/fonc.2018.00254)

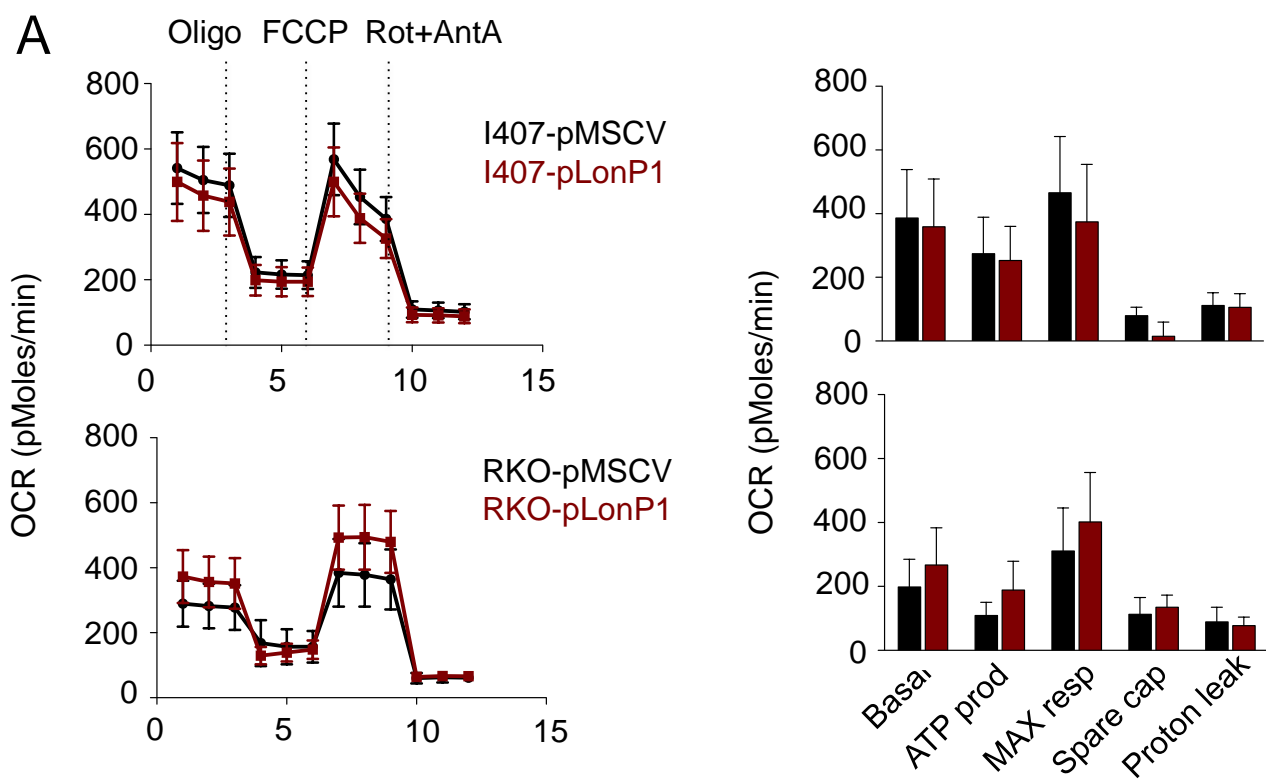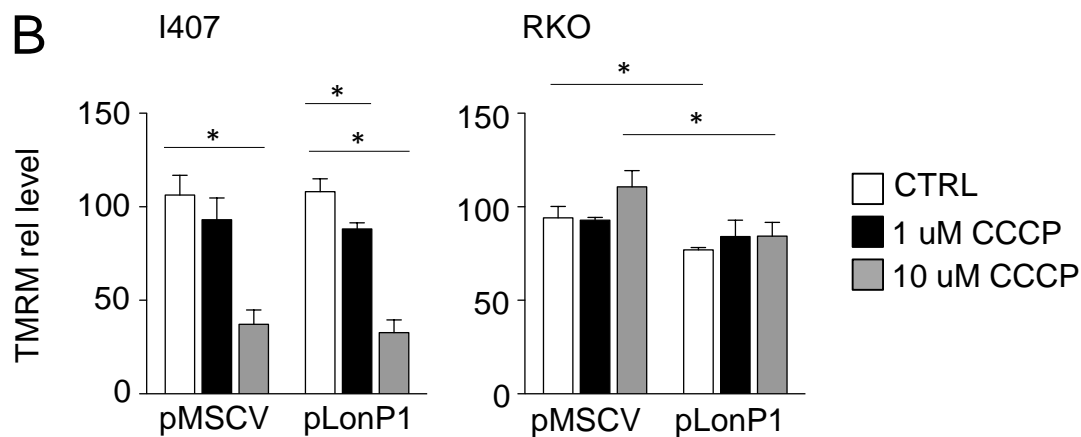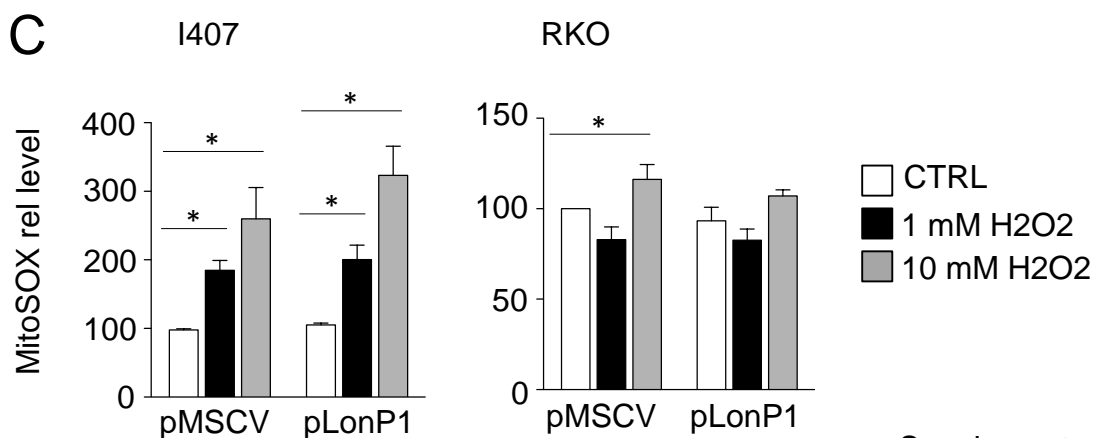

Supplementary Figure 2

Supplement: Figure S2 — LonP1 slightly modulates mitochondrial activity in I407 and RKO colon cancer cells. (A) Representative traces and quantitative analysis of the oxygen consumption rate in indicated cells. Subsequent injections of oligomycin (Oligo), mitochondrial decoupler carbonyl cyanide-4-(trifluoromethoxy)phenylhydrazone (FCCP), complex I inhibitor rotenone (Rot) and complex III inhibitor (AntA) were performed as indicated. (B) Mitochondrial membrane potential quantification as assayed by tetramethyl rodhamine methyl esther (TMRM) in the presence or absence of CCCP in indicated cells. Data are expressed as percentage of increase in median fluorescence intensity (MFI) and represented the mean ± SD (n = 3); *P < 0.05. (C) Mitochondrial anion superoxide quantification as assayed by MitoSOX Red Mitochondrial Superoxide Indicator (mitoSOX) in the presence or absence of hydrogen peroxide in indicated cells. Data are expressed as percentage of increase in MFI and represented the mean ± SD (n = 3); *P < 0.05. [file image_2.PDF]
