# Supplementary material for: LonP1 Differently Modulates Mitochondrial Function and Bioenergetics of Primary Versus Metastatic Colon Cancer Cells
Source: Front Oncol. 2018 Jul 9;8:254. doi: 10.3389/fonc.2018.00254 (PMC6046640; doi:10.3389/fonc.2018.00254)
Supplement: Figure S1 — LonP1 modulates E-cadherin, N-cadherin, and β-ctn in I407 and RKO cells. (A) Representative Western blot analysis and relative protein level of LonP1, β-catenin (β-ctn), E-cadherin (E-cad), N-cadherin (N-cad) in I407 cells overexpressing LonP1 (namely pLonP1) and in control cells, i.e., cells stably transfected with empty vector (namely pMSCV). β-actin was used as loading control. Densitometries are reported in histograms, and data are reported as mean ± SD (n = 3). *P < 0.05 and **P < 0.01. (B) Representative phase contrast images of I407 cells overexpressing LonP1 (pLonP1) and control cells (pMSCV). Scale bar, 10 µm. (C) Representative Western blot analysis and relative protein level of LonP1, β-catenin (β-ctn), E-cadherin (E-cad), and N-cadherin (N-cad) in RKO cells overexpressing LonP1 (namely pLonP1) and in control cells, i.e., cells stably transfected with empty vector (namely pMSCV). β-actin was used as loading control. Densitometries are reported in histograms, and data are reported as mean ± SD (n = 3). *P < 0.05 and **P < 0.01. [file image_1.PDF]

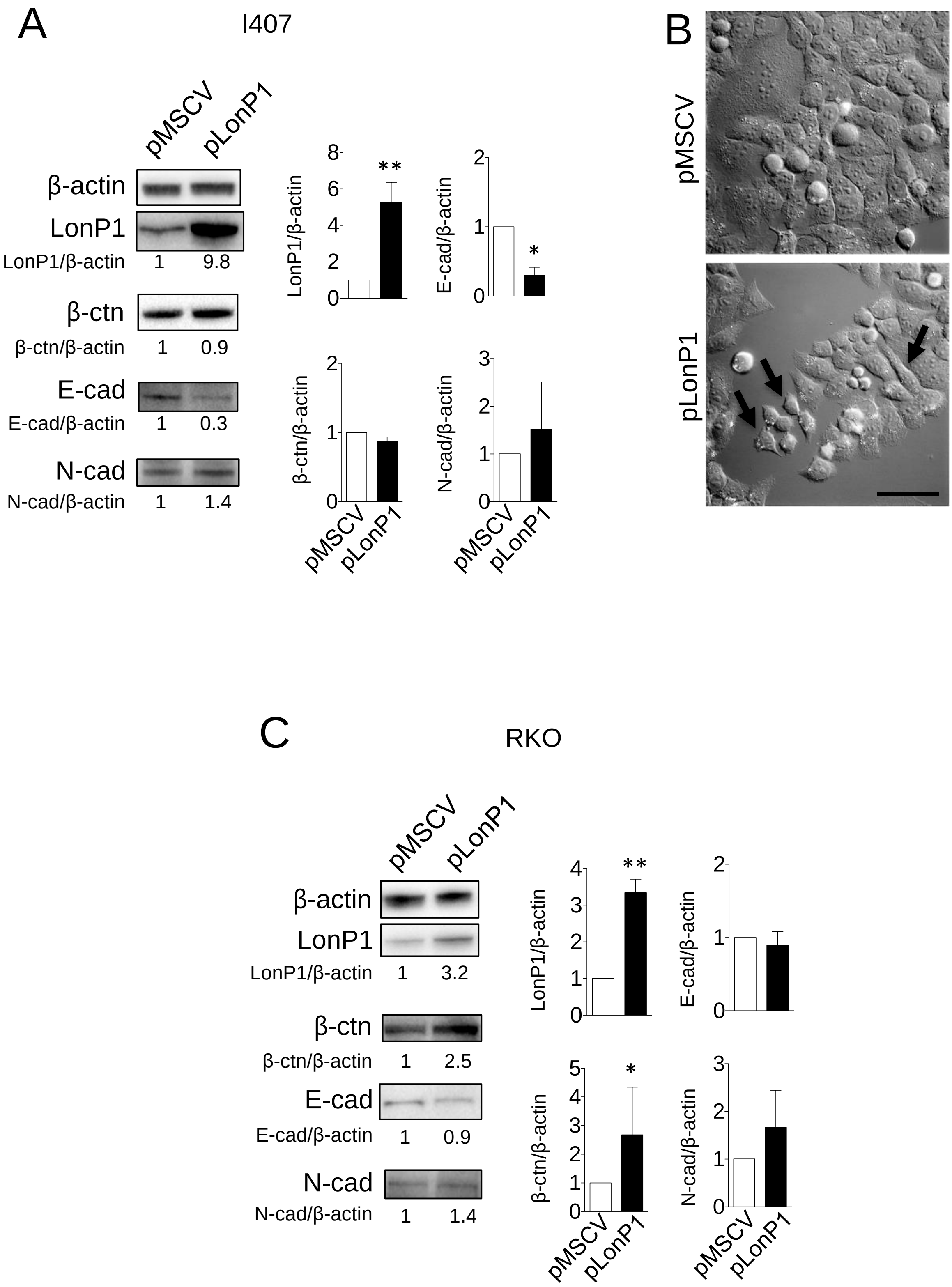

Supplementary Figure 1
